# Integral correlation for uneven and differently sampled data, and its application to the Law Dome Antarctic climate record

**DOI:** 10.1038/s41598-020-74532-9

**Published:** 2020-10-15

**Authors:** Jason L. Roberts, Lenneke M. Jong, Felicity S. McCormack, Mark A. Curran, Andrew D. Moy, David M. Etheridge, Jamin S. Greenbaum, Duncan A. Young, Steven J. Phipps, Wenyue Xue, Tas D. van Ommen, Donald D. Blankenship, Martin J. Siegert

**Affiliations:** 1 Australian Antarctic Division, Kingston, TAS 7050 Australia; 2 Australian Antarctic Program Partnership, Institute for Marine and Antarctic Studies, University of Tasmania, Hobart, TAS 7004 Australia; 3 Monash Ice Sheet Initiative, School of Earth, Atmosphere and Environment, Monash University, Clayton, VIC 3168 Australia; 4 Institute for Marine and Antarctic Studies, University of Tasmania, Hobart, TAS 7001 Australia; 5 Climate Science Centre, CSIRO Oceans and Atmosphere, PMB 1, Aspendale, VIC 3195 Australia; 6 Scripps Institution of Oceanography, University of California San Diego, La Jolla, CA 92093 USA; 7 Institute for Geophysics, Jackson School of Geosciences, University of Texas at Austin, Austin, TX 78712-1692 USA; 8 Department of Earth Science and Engineering, Grantham Institute, Imperial College London, South Kensington, London, SW7 2AZ UK

**Keywords:** Cryospheric science, Computational science

## Abstract

We present a new simple and efficient method for correlation of unevenly and differently sampled data. This new method overcomes problems with other methods for correlation with non-uniform sampling and is an easy modification to existing correlation based codes. To demonstrate the usefulness of this new method to real-world examples, we apply the method with good success to two glaciological examples to map the ages from a well-dated ice core to a nearby core, and by tracing isochronous layers within the ice sheet measured from ice-penetrating radar between the two ice core sites.

## Introduction

Although data correlation is a keystone of quantitative analysis, its application to many data series can be problematic. The ubiquitous Pearson correlation coefficient is defined for two data series with identical (although possibly uneven) sampling. However, different (and possibly uneven) sampling between data series is a common feature in many fields of study, requiring alternatives to directly applying the Pearson correlation.

When dealing with differently and unevenly sampled data, the Pearson correlation may introduce biases^1^, especially if interpolating across large (relative to the periods of the underlying signals) data-gaps. More sophisticated approaches include: resampling autoregressive series onto an evenly sampled base and using standard methods for uniformly sampled data (BINCOR)^2^, using the relationship between correlation and the power spectrum (Wiener–Khinchin theorem^3^) and methods for estimating the power spectrum for unevenly sampled data (e.g. Lomb-Scargle periodograms); ignoring small miss-matches in data spacing (so-called correlation slotting), which is equivalent to using a rectangular kernel; and more computationally expensive kernels including sinc and Gaussian functions. Rehfeld et al.^4^ provides a good overview of these methods, and conclude that for many applications Gaussian kernel correlation out-performs the other methods, especially when the data spacing is skewed^4^. However, this out-performance by Gaussian kernel correlation does not guarantee accurate results, and more robust methods are required.

Here we present a new method for calculating the correlation between two differently and unevenly sampled series *x*(*t*) and *y*(*t*), which we refer to as a Segmented Linear Integral Correlation Kernel (SLICK) correlation. The method is based on sub-dividing the domain into potentially disconnected regions based on the data density. Within each sub-domain we apply linear interpolation and evaluate the contribution to the correlation using analytic integration. This last step accurately weights the contribution to the correlation by the segment length. SLICK correlation is computationally inexpensive while out-performing Gaussian kernel correlation over a range of test problems; we describe the details of SLICK next, then apply the method to both test and real-world examples.

## Methods

### Existing methods

For two identically sampled data series (*x* and *y*) of *n* points each, the Pearson correlation coefficient ($r_P$) is given by:

1 $$\begin{aligned} r_P=\frac{\sum _{i=1}^{n}\left( x_i-\bar{x}\right) \left( y_i-\bar{y}\right) }{\sqrt{\sum _{i=1}^{n}\left( x_i-\bar{x}\right) ^2\sum _{i=1}^{n}\left( y_i-\bar{y}\right) ^2}}, \end{aligned}$$

where $\bar{x}$ and $\bar{y}$ are the average values for the *x* and *y* series respectively.

For unevenly and differently sampled data we can use Gaussian kernel correlation^4^ modified for a more consistent treatment of the data series mean and variance^1^. Specifically, the correlation $r_G$ between two data series *x* and *y* of lengths *n* and *m* respectively, is given by:

2 $$\begin{aligned} r_G=\frac{1}{\sigma _x\sigma _y}\frac{\sum _{i=1}^n\sum _{j=1}^m\left( x_i-\bar{x}\right) \left( y_j-\bar{y}\right) \kappa \left( d_{x_i}-d_{y_j}\right) }{\sum _{i=1}^n\sum _{j=1}^m\kappa \left( d_{x_i}-d_{y_j}\right) }, \end{aligned}$$

where $d_x$ and $d_y$ are the independent variables (basis) for the series *x* and *y* respectively, and $\kappa (d)=\frac{1}{\sqrt{2\pi h}}\exp (-d^2/2h^2)$ is the Gaussian kernel with width parameter *h* (typically 0.25). The series standard deviations $\sigma _x$ and $\sigma _y$ are calculated using the same Gaussian kernel weighting as for $r_G$.

### Segmented linear integral correlation kernel (SLICK)

Here, we detail SLICK correlation between two differently and unevenly sampled series *x*(*t*) and *y*(*t*). The method considers the combined domain of the two series, and then divides this domain into sub-domains each within some distance *h* of a data point. Details on the selection of *h* will be given later. Any sub-domain that does not contain data from both series is discarded, leaving $n_{valid}$ valid sub-domains. This alleviates the main problem with simply linearly interpolating one data set onto the basis of the other series, namely interpolating across large data gaps. Linear interpolation is carried out on both data series over each sub-domain, allowing for $n\rightarrow \infty$ data points in each sub-domain. The SLICK correlation $r_{SLICK}$ can be calculated as:

3 $$\begin{aligned} r_{SLICK}=\frac{\sum _{j=1}^{n_{valid}}\sum _{i=1}^{n}\left( x_i-\bar{x}\right) \left( y_i-\bar{y}\right) }{\sqrt{\sum _{j=1}^{n_{valid}}\left( \sum _{i=1}^{n}\left( x_i-\bar{x}\right) ^2\sum _{i=1}^{n}\left( y_i-\bar{y}\right) ^2\right) }}. \end{aligned}$$

Finally, replacing the $n\rightarrow \infty$ sums with integrals gives:

4 $$\begin{aligned} r_{SLICK}=\frac{\sum _{j=1}^{n_{valid}}\int \left( x(t)-\bar{x}\right) \left( y(t)-\bar{y}\right) dt}{\sqrt{\sum _{j=1}^{n_{valid}}\left( \int \left( x(t)-\bar{x}\right) ^2dt\int \left( y(t)-\bar{y}\right) ^2dt\right) }}, \end{aligned}$$

which can be evaluated analytically for linear interpolation.

There is one free parameter *h*, which defines how closely data from the two series must be to be included in the calculation. While it is possible to tune this value for a given problem type, we have found that a value of $0.4\times \max \left( median_x,median_y, IQR_x, IQR_y\right)$ performs well over a broad range of problems, where $median_x$ is the median data spacing for series *x* and $IQR_x$ is the inter-quartile range of the data spacing for series *x*. This form for *h* allows the selection to be sensitive to both the spacing and skewness of the data. Lower values of *h* would result in more data being discarded, while larger values would result in interpolation across larger data gaps.

## Results

### Test cases

We consider several test cases to assess the performance of the SLICK algorithm compared to Gaussian kernel correlation. We exclude Pearson correlation from the test cases as Gaussian kernel correlation has been shown to out-perform it^4^. The first test case is for piece-wise linear functions, inspired by one of the authors having difficulty with Gaussian kernel correlation when analysing ice-penetrating radar data with signals of this general form. The second example is the correlation between trigonometric functions, which are common in many areas of study. To allow for direct comparison between our new method and Gaussian kernel correlation, we then consider two examples from Rehfeld et al.^4^ using functional forms common to climate studies, two autoregressive cases and sinusoids with random phase, where Gaussian kernel correlation performs well compared to other methods.

#### Piecewise linear

Consider the piecewise linear ramp function, with the step occurring over a domain of length 0.1, given by:

5 $$\begin{aligned} f(x)=\left\{ \begin{array}{ll} 0&{} \hbox {if } 0<x<4.9\\ 10(x-4.9)&{} \text{ if } 4.9\le x\le 5\\ 1&{} \text{ if } 5.0<x<10. \end{array} \right. \end{aligned}$$

**Figure 1:**
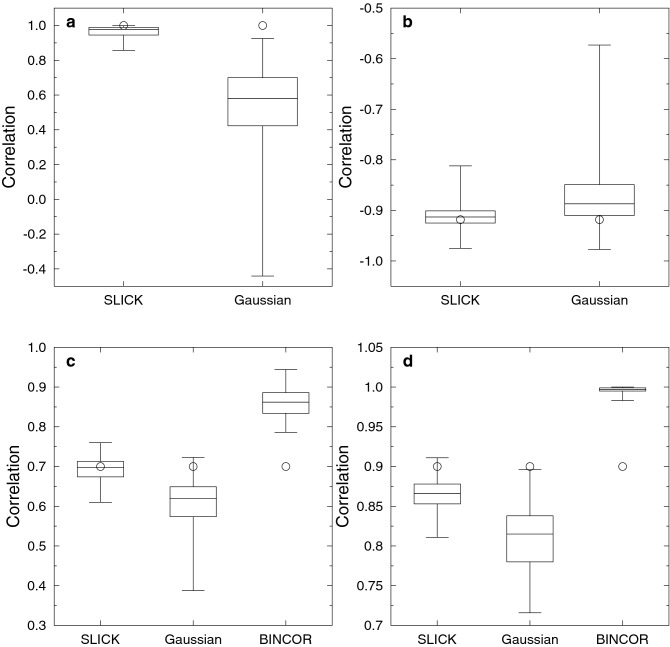
Box-whisker plots show the ensemble extreme correlations and the ensemble quartile correlations. The theoretical correlation is shown by the circle. (**a**) Piecewise linear case (note, even the worst SLICK correlation is better than more than 75% of the Gaussian kernel result). (**b**) Trigonometric functions case. (**c**) Autoregressive case for lag-1 auto-correlation of 0.7. (**d**) Autoregressive case for lag-1 auto-correlation of 0.9.

We generate time series by resampling the above function using uniform random sampling, with a total of 11 points, at least two of which must lie in the range [4.9,5.0]. The correlation coefficient for any two time series should be 1. To gather statistics on the performance of SLICK, we repeat the process for an ensemble of 100 members and compare the results to Gaussian kernel correlation (Fig. 1a).

#### Trigonometric functions

Consider the Pearson correlation coefficient between the two functions $\cos (x)$ and $\sin (x)$ over the interval $(0,\pi /2)$. The expected Pearson correlation coefficient between these two series can be estimated by discrete sampling in the limiting case of an infinite number of discrete samples:

6 $$\begin{aligned} \frac{1/2-2/\pi }{\left( \pi /4-2/\pi \right) } \approx -0.918. \end{aligned}$$

We evaluate the accuracy of two different algorithms for unevenly and differently sampled data and compare to the classic algorithm for evenly and identically sampled data in Fig. 1b. To ensure results consistent with Eq. 6 we explicitly include the points at the range extremes (i.e. 0 and $\pi /2$). To evaluate how robust the estimates are, we repeat the calculations for 1000 ensemble members, using uniform random resampling, and show the mean, standard deviation and extreme values for the ensembles (Fig. 1b).

#### Autoregressive

Consider an AR(1) autoregressive function typical of many climate processes, as follows:

7 $$\begin{aligned} X(t_{i+1})=\exp \left( -\frac{t_{i+1}-t_i}{\ln \phi }\right) X(t_i)+\epsilon _i, \end{aligned}$$

where $\phi$ is the AR(1) coefficient and $\epsilon _i$ uncorrelated Gaussian distributed noise.

The autocorrelation at a lead or lag of one time unit should equal $\phi$. We test this using a 100 member ensemble with each member being a time series 1000 years in duration, sampled in time with a Gamma-distributed^5^ interval with mean one year and skewness 2.85.

The results are shown in Fig. 1c,d. For the smaller lag-1 autocorrelation of 0.7 more than 50% of the SLICK results are within 0.026 of the correct result, while 50% of the Gaussian kernel correlation results are in error by more than 0.081. For the larger lag-1 auto-correlation of 0.9, SLICK still outperforms Gaussian kernel correlation, with more than 75% of the SLICK results better than the best 25% from Gaussian kernel correlation. However, the SLICK results are not as good as for the lower lag-1 auto-correlation. In this case the correct result is in the highest quartile of SLICK results, but this is better than Gaussian kernel correlation where the range of the results does not span the correct value. We also show the results from BINCOR^2^ for this test case. BINCOR resamples two autoregressive series onto identical and evenly sampled basis and then applies standard methods for uniformly sampled data. BINCOR over-estimates the correlation for both the autoregressive test cases, with the range of the ensemble estimates not including the correct value.

#### Sinusoids with random phase

The final test case is the sum of three sinusoids of equal amplitude, with random phase and periods of 18, 21 and 41 years respectively. The epoch is 1000 years and the average sampling rate four years, with varying degrees of Gamma-distributed skew. Again we use a 100 member ensemble, with random resampling, to evaluate the performance of SLICK correlation.

**Figure 2:**
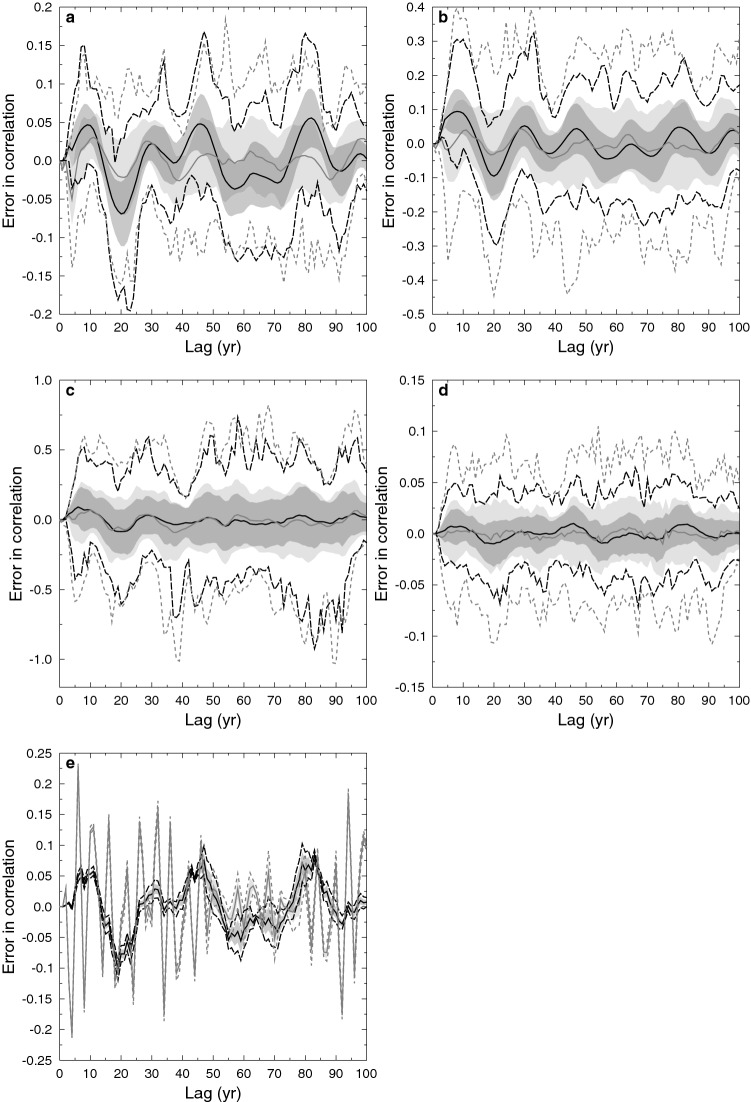
Error in auto-correlation as a function of lag for the random phase sinusoidal case showing SLICK (dark solid and dotted lines and shading) and Gaussian kernel (light solid and dashed lines and shading), median (solid line), inter-quartile range (shading) and extreme (dotted and dashed lines) for a 100 member ensemble. Skewness are (**a**) 1.0, (**b**) 2.85 and (**c**) 5.0. (**d**) Higher mean sampling rate of 1 year (compared to 4 years for **a**–**c** and **d**) and skew of 2.85. (**e**) Uniform sampling. Note different y-axis scales.

The low-frequency structure (with a period around 15–20 years in the lag) seen in the SLICK median and inter-quartile range (Fig. 2) is due to the insufficient sampling rate compared to the beat frequencies of the sinusoids and is largely eliminated by a higher sampling rate (Fig. 2d).

Because SLICK uses a more compact stencil than the Gaussian kernel, it performs better in the case of uniform sampling. This is exemplified by the random phase sinusoid case discussed above where the sampling has a uniform interval of four years. While structure in the error is evident for SLICK (Fig. 2e) associated with under-sampling of the beat frequency, overall the errors are much smaller than for the Gaussian kernel.

#### Summary

For all the test cases, SLICK showed greatly reduced variability in the estimated correlation coefficient, and in general a much more accurate median result. The only exceptions to this were for some lags when calculating the auto-correlation for the sinusoids with random phase. For these cases the insufficient sampling rate compared to the beat frequencies and the inability of the Gaussian kernel method to correctly follow the low frequency structure associated with this under-sampling, resulted in anomalously good results for Gaussian kernel correlation.

### Glaciological examples

#### Dating a shallow Antarctic Ice Core

Here we date a high-resolution ice core (DE08) from Law Dome, East Antarctica by correlating down core stable water isotope (oxygen isotope $\delta ^{18}$O) measurements against the nearby well-dated Dome Summit South (DSS) ice core. Stable water isotopes have a well defined seasonality at Law Dome^6^, and when combined with other seasonally varying indicators, allows us to achieve annual temporal resolution at both sites.

**Figure 3:**
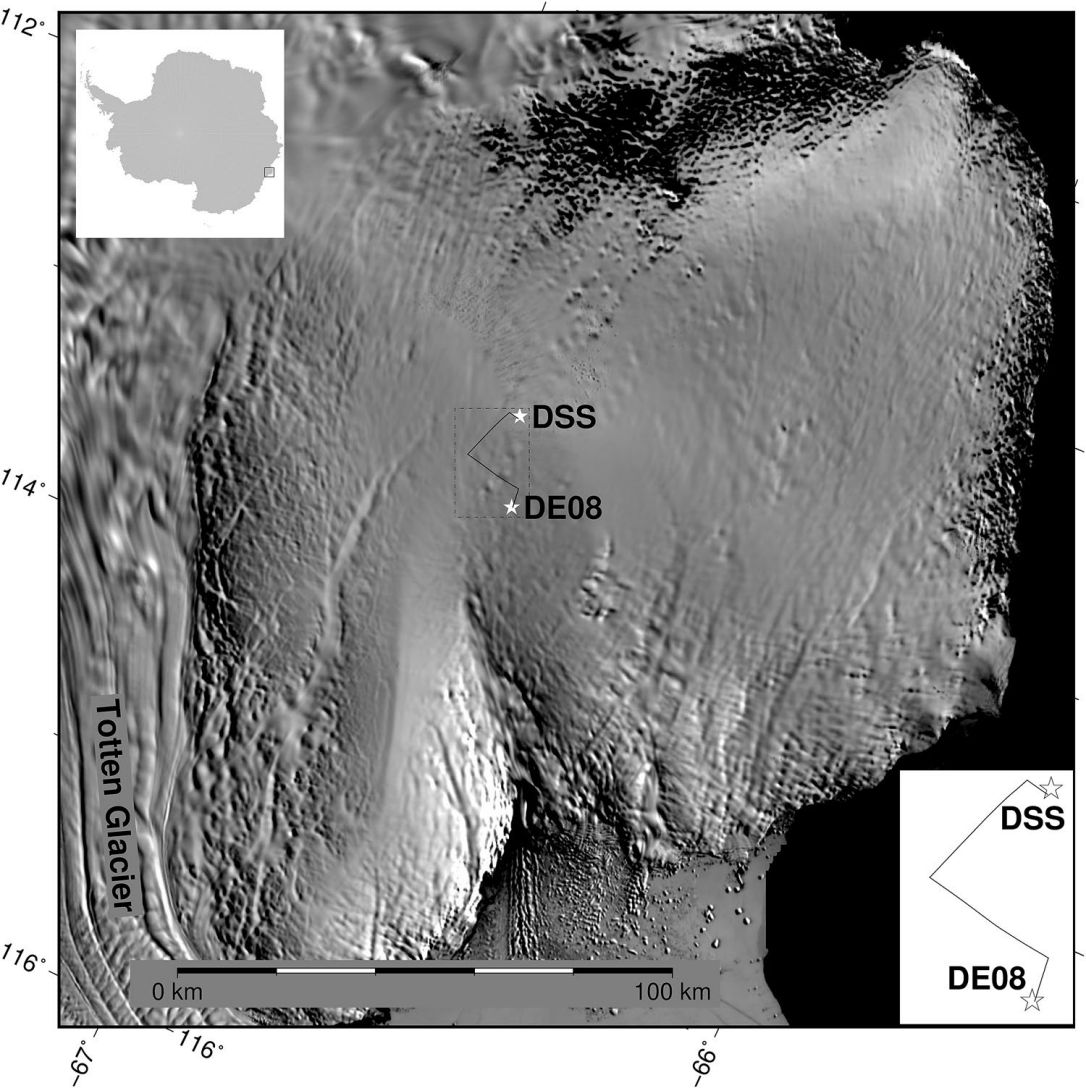
Law Dome, East Antarctica, showing the location of the two ices cores (white stars) and the 4 radar profiles (black lines) joining the two ice core sites. Upper left insert shows location of main panel, lower right insert shows enlarged view of the location of ice core sites and radar profiles.

Law Dome is a small independent ice cap in coastal East Antarctica, with a strong orography driven snow accumulation gradient and sufficient snowfall near the dome summit for annual temporal resolution^7^. As there is no long-term snowfall record for DE08 independent of the ice core record, we use the fact that atmospheric modelling and reanalysis shows that snowfall across Law Dome is highly spatially correlated^7^ to map the age scale from the DSS main ice core^8^ to the DE08 ice core, which was drilled 16 km to the east (see Fig. 3).

When calculating snow accumulation rates from the depth of annual horizons, several corrections to the depths must be made. First, as the firn compresses under its own weight, and therefore changes density, all depths are corrected for the equivalent overburden mass of snow being at the density of glacial ice (917 kg m$^{-3}$) and all depths reported as “ice-equivalent depths” (IE). Second, differential vertical motion of the ice results in a vertical strain rate and thinning of annual layers over time as they advect deeper into the ice sheet.

We use a linear model to map the depths of $\delta ^{18}$O records between DSS (n = 3125) and DE08 (n = 1864) allowing for an offset and linear scale factor, the latter of which represents the ratio of the long term average snow accumulation rates at the two sites. To allow for shorter term variations between the two sites (due possibly to the movement of sastrugi across the ice core site) we allowed for a fine-scale correction to the depth of individual $\delta ^{18}$O measurements of up to 25 mm compared to the depth of neighbouring measurements. To optimise the linear model and fine-scale corrections, we systematically vary these parameters to maximise the Pearson correlation coefficient (r = 0.827) calculated using SLICK. As SLICK does not require either even or the same sampling between the two series, the application of the linear model with fine-scale corrections only requires a recalculation of the sample depth, not linear interpolation of the data which may cause biases^1^.

**Figure 4:**
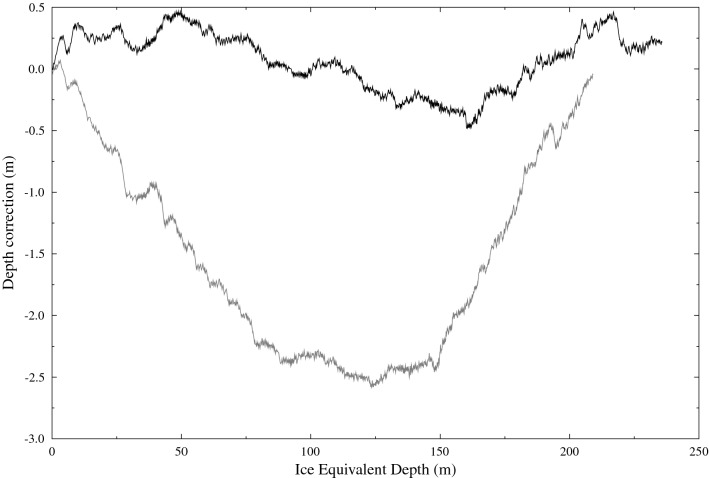
Fine scale depth correction for mapping DSS to DE08 ice cores. The effect of correction for vertical strain rate can be seen, with no vertical strain rate correction (grey line) and vertical strain rates of $6.57\times 10^{-4}$ year$^{-1}$ and $1.221\times 10^{-3}$ year$^{-1}$ for DSS and DE08 respectively (black line).  Notice the greatly reduced scale of the depth correction when vertical strain rate corrections are applied.

In the absence of any additional data, one method to correct for the vertical strain-rate-induced layer thinning is to assume no long term trend in the snow accumulation rate. However, this assumption is likely to be incorrect for moderately short duration records. Rather than base our vertical strain rate corrections on a relatively short (order 180 years) record, we destrained the DSS record using the strain rate of $6.57\times 10^{-4}$ year$^{-1}$, based on a 2000 year record^7^, and estimated the DE08 strain rate by optimising the fine scale correction. In particular, when the corrections for the DSS and DE08 strain rates are incorrect (or not applied at all), there will be large scale structure in the fine scale correction, while a consistent combination of strain rates will result in small corrections with little large scale structure. For example, in Fig. 4 the influence of correcting for vertical strain rate is clearly visible.

**Figure 5:**
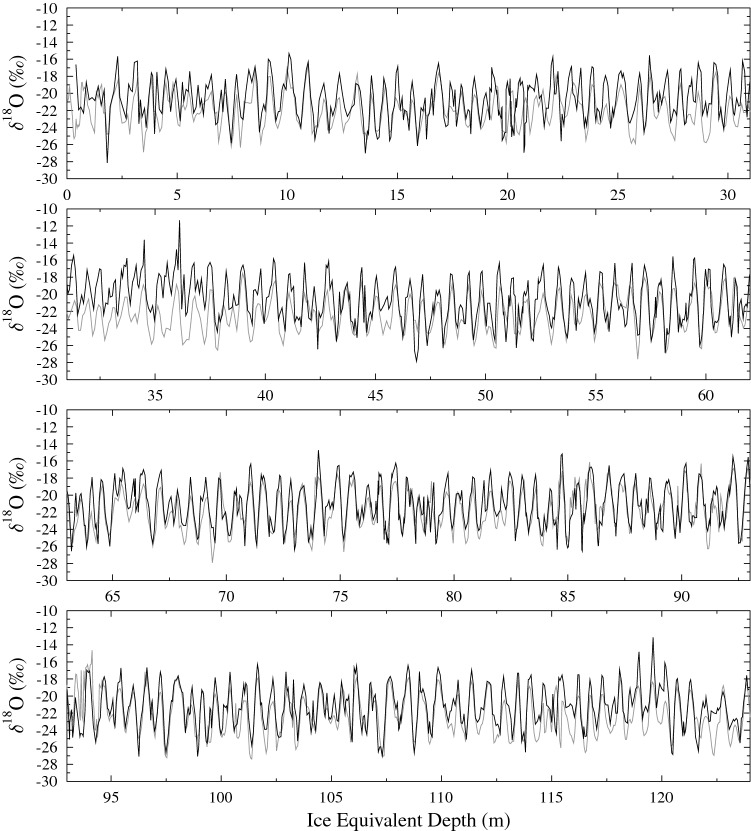
Stable water isotope (oxygen isotope $\delta ^{18}$O) record for DSS (grey) and depth remapped DE08 (black).  The correlation between these two records is r = 0.827.

We find the combination of a vertical strain rate of $1.221\times 10^{-3}$ year$^{-1}$ and a linear scale factor of 0.52335 provides the smallest fine scale depth correction with acceptable correlation between DSS and DE08. Combined with the current best estimate of the long term snow accumulation rate of 0.686 m year$^{-1}$ IE for DSS^7^ gives an estimated long term snow accumulation rate at DE08 of 1.311 m year$^{-1}$ IE. This corresponds to 1202 kg m$^{-2}$ year$^{-1}$ which is 8.5% higher than the previous estimate^9^. The estimated vertical strain rate is consistent with what we would expect compared to DSS, in particular scaling the DSS vertical strain rate by the ratio of the snow accumulation rates, and noting very similar estimated ice thicknesses at the two sites, gives an estimated vertical strain rate at DE08 of $1.255\times 10^{-3}$ year$^{-1}$ less than 3% larger than our optimised value. The comparison between the DSS and remapped DE08 record is shown in Fig. 5.

#### Tracing internal layers within the Antarctic Ice Sheet

To verify that the inferred snow accumulation rate at DE08 is reasonable, we use the SLICK correlation method to trace internal layers in ice penetrating radar data in this region. Internal layers in the ice sheet are known to be due to changes in ice chemistry associated with changes in atmospheric composition (such as changes in atmospheric sulphur content associated with volcanic eruptions), and therefore can represent isochrones for the deposition of snow at the surface^10^. The subsequent advection of these isochrones to depth is primarily a function of the local snow accumulation rate and variations in ice sheet dynamics. Over relatively small spatial and temporal scales the ice sheet dynamics can be assumed to be fairly constant, and relative changes in depth of isochrones is driven by local differences in snow accumulation rates^10^.

We trace an internal layer from aerogeophysical surveys, including ice-penetrating radar, from the ICECAP program^11,12^. A complete radar profile between DSS and DE08 is constructed from 4 flight segments (ASB/JKB1a/GL0211a^13^ ASB/JKB2d/1973La, ICP3/JKB2d/F52T01a and ASB/JKB2d/F50T01a^14^), levelled to constant elevation, stitched together at their crossover points and the resulting image enhanced using an unsharp mask to improve the contrast. The horizontal resolution is typically around 22.5 m and the vertical resolution in the ice is around 1.69 m based on a radar propagation speed of 169 m $\upmu$s$^{-1}$ in ice and ignoring firn effects. To allow for interpretation of features in the radar signal in terms of the ice core chemistry, we convert the radar signal from the time domain into a depth domain. As SLICK does not depend on the sampling basis (only the relative sample spacing), this conversion to a depth domain does not change the correlations.

**Figure 6:**
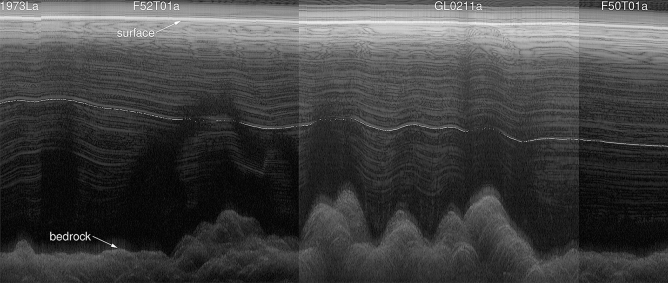
Radar profile between DSS (left of image) to DE08 (right of image).  The traced internal layer corresponds to the 1257 CE series of volcanic eruptions.  Radar profiles from 4 different flights are stitched together (flight segments are labeled at the top of the image).  Horizontal distance is approximately 33.75 km and scale is exaggarated 10 times in the vertical.

A strong internal reflector at approximately 435 m depth at DSS is traced (Fig. 6) by optimising the SLICK correlation against a reference reflector for small vertical windows (approximately 54 m in size) centered around the reflector for individual vertical slices through the radargram. A small scale factor (0.98–1.02) and a vertical offset (− 2.54 to 2.54 m per vertical slice) is allowed for. A local correlation in excess of a threshold (r = 0.85) results in a “match” and an update in the reference reflector (updated to 95% of the current reference reflector and 5% of the window from the current vertical slice), while a local correlation below this will result in skipping this vertical slice and searching over a larger vertical offset.

The layer chosen at around 435 m at DSS corresponds to the 1257 CE Samalas volcanic eruption^8,15^, measured at a depth around 418 m in the DSS ice core relative to the 1988 ice surface. Approximately 9 m of this difference in depth is due to downward advection since 1988 and another 9 m due to the increased radar propagation velocity in the less dense firn compared to glacial ice. The internal layer has a depth of 597 m at DE08, and correcting both sites for the vertical strain rates of 6.57$\times 10^{-4}$ year$^{-1}$ and 1.255$\times 10{-3}$ year$^{-1}$ for DSS and DE08, respectively, gives the effective linear scale factor of 0.606. Due to the gradient in snow accumulation across Law Dome, and the horizontal motion of ice away from the dome summit, resulting in deeper ice at DE08 originating closer to the dome summit (and hence at lower snow accumulation site) we would expect the snow accumulation ratio to be larger than we found using the ice core records.

**Figure 7:**
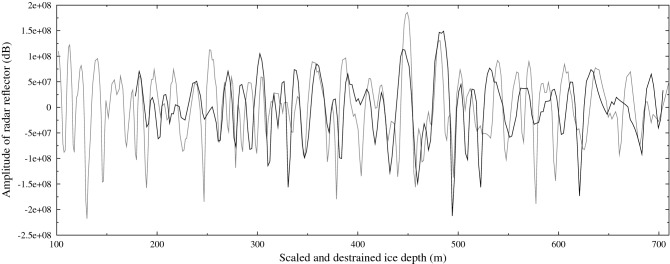
Radar reflection profiles at DSS (black) and DE08 (grey). Depths have been destrained at both sites and rescaled at DE08 by the inverse ratio of the snow accumulation rates (0.544).

To treat the case where internal layers are difficult to trace over the entire distance between the two sites of interest (possibly due to aircraft roll or snow surface features), we also estimate the snow accumulation rate and vertical strain-rate at DE08 by optimising the correlation between the radar profiles at these two sites. The dominant feature of the radar profiles is a decrease in the returned power due to attenuation, which we remove by high-pass filtering both signals using a Gaussian filter with a half-power bandwidth of 10 m. As the two sites have different snow density profiles with depth, and hence different propagation speeds of radar signals, we have optimised the signals over the depth range 169–704 m (using a radar propagation speed of 169 m $\upmu$s$^{-1}$) (see Fig. 7). The optimal correlation (r = 0.385) is for an accumulation rate of 1156 kg m$^{-2}$ year$^{-1}$ and a vertical strain rate of $1.256\times 10^{-3}$ year$^{-1}$ at DE08. The estimated snow accumulation rate is slightly smaller than the estimate from the ice core records, but includes older ice, which would have originally been deposited closer to DSS (and has advected downstream over time), so we would expect a lower estimated snow accumulation rate at depth.

## Discussion

SLICK correlation overcomes the problem of correlation on differently and unevenly sampled data by sub-dividing the domain into potentially disconnected regions with sufficient data density to allow for the accurate use of linear interpolation. By not interpolating over large data gaps, the method avoids the main source of error associated with applying a more traditional continuous domain linear interpolation. Calculation within each linear segment is via analytic integration which automatically weights the overall result by the segment length. While higher order functions could have been used within sub-domains, this raised three issues. First, it would require more data points from both series to be within a suitable window, probably reducing the number of valid segments. Second, higher order interpolation is subject to Runge‘s phenomenon^16^. Third, for many applications Trapezoidal integration (linear interpolation) is more accurate than Simpson’s rule (based on quadratic interpolation) for narrow peak-like functions^17^.

SLICK correlation outperforms Gaussian correlation in all of the test cases, with greatly reduced variability (as measured by the inter-quartile range) and improved median estimates. For the piecewise linear test case, the worst estimate using SLICK correlation is within 15% of the correct answer, while for Gaussian kernel correlation 75% of the results have an error in excess of 30% and the worst result is in error by 144%. Even for the trigonometric test case, where the functions are varying much more smoothly than for the piecewise linear test case, SLICK correlation performs substantially better. The inter-quartile range for SLICK correlation contains the correct result and the inter-quartile range differs from the correct result by − 0.007 to 0.017, while the correct result is outside the inter-quartile range for Gaussian kernel correlation, with errors in the range 0.008–0.069.

SLICK correlation also out-performs Gaussian kernel correlation for the two test cases of Rehfeld et al.^4^. Again, for the autoregressive test cases, the inter-quartile range for SLICK correlation contains the correct result, while it lies outside the inter-quartile range for Gaussian kernel correlation. Gaussian kernel correlation performs better in the random phase sinusoids test case, although SLICK correlation still outperforms it. Errors associated with under-sampling of the beat frequency are more clearly shown with SLICK correlation, due to the smaller inter-quartile range. This structure becomes less evident with increasingly skewed sampling, as increasing skew results in better high-frequency coverage, while uniform sampling highlights the merits of SLICK correlation.

SLICK correlation was applied to two real-world glaciological examples to allow for the interpretation of relatively sparse information at the DE08 site leveraging the much more extensive knowledge at the DSS site, on Law Dome, East Antarctica. Non-linear remapping of the data was needed to account for firn compaction, vertical and horizontal advection post deposition and differing radar propagation velocity in the less dense firn compared to glacial ice. This remapping was achieved through application of SLICK correlation to small sub-domains of the data. The depths in each sub-domain were linearly distorted to maximise the SLICK correlation, and the combined effects of these linear distortions over sub-domains, when applied to the entire domain, provided the non-linear remapping required. For these two applications, the actual values from the SLICK correlation were not the key result, SLICK correlation provided the cost-function that was maximised to allow for a useful mapping of data between the two sites, in this case the long term accumulation rates.

For the case studied, SLICK correlation provides an accurate and robust estimate of the correlation between two differently and unevenly sampled data series. The variability in correlation estimates is reduced compared to Gaussian kernel correlation, and the method is computationally efficient as there is no need to calculate the computationally expensive exponential function within a double nested loop. This computationally efficient and robust method is applicable to many real world problems with either missing data from an otherwise uniformly sampled series (e.g. astronomy, rainfall, streamflow, or temperature time-series) or where uniform sampling is difficult (e.g. speleothem, coral and ice core climate proxy studies). Fortran, Matlab and Python versions implementing SLICK correlation are freely available at https://github.com/jlr581/SLICK_correlation.
